# A collaborative multi-view fusion framework with Deep Forest for multi-class diagnosis of Alzheimer’s disease

**DOI:** 10.3389/fnagi.2026.1888587

**Published:** 2026-09-24

**Authors:** Yanhong Luo, Nan Dai, Yu Qiao, Tingting Zheng, Yan Zhang, Yanbo Zhang, Hongmei Yu, Hongyan Cao, Aiai Zhang, Mengyuan Li, Yongao Li, Na Yang, Tianyi Wang, Jie Zhou, Xiaoyan Li

**Affiliations:** 1 Shanxi Key Laboratory of Biomedical Data and Statistics, Department of Health Statistics, Shanxi Medical University, Jinzhong, China; 2 Key Laboratory of Coal Environmental Pathogenicity and Prevention (Shanxi Medical University), Ministry of Education, Taiyuan, China; 3 Academy of Medical Sciences, Shanxi Medical University, Taiyuan, Shanxi, China; 4 Department of Nuclear Medicine, Shanxi Province Cancer Hospital/Shanxi Hospital Affiliated to Cancer Hospital, Chinese Academy of Medical Sciences/Cancer Hospital Affiliated to Shanxi Medical University, Taiyuan, China; 5 Department of Pulmonary and Critical Care Medicine, Shanghai University of Medicine & Health Sciences Affiliated Zhoupu Hospital, Shanghai Pudong New Area Zhoupu Hospital, Shanghai, China

**Keywords:** Alzheimer’s disease, collaborative learning, data fusion, Deep Forest, multi-class classification

## Abstract

**Objective:**

A key aspect of diagnosing Alzheimer’s disease (AD) is identifying mild cognitive impairment (MCI), a sensitive transitional stage between normal cognition (NC) and AD. We divided AD disease progression into three stages: NC, MCI, and AD according to severity. We used CoopLearning to integrate multi-view medical data with a Deep Forest (DF) classifier to classify three stages of AD to aid diagnosis.

**Methods:**

We used data from the Alzheimer’s Disease Neuroimaging Initiative database. The research variables were divided into five views: demographic information, biological sample information, relevant scale information, pixel identification, and magnetic resonance imaging regional information. We compared CoopLearning for feature selection and fusion, least absolute shrinkage and selection operator cross-validation (LassoCV) for single-view feature selection, and LassoCV for multi-view feature selection. The fused data were processed using Synthetic Minority Oversampling Technique (SMOTE), SMOTETomek, and SMOTE Edited Nearest Neighbors (ENN) methods to achieve class balance. The balanced data were then applied to multiple classifiers, such as DF, Random Forest, etc., for performance evaluation.

**Results:**

Multi-view feature selection and fusion using CoopLearning, multi-view feature selection, and single-view overall feature selection using LassoCV identified 20 features from five views. Applying these results to the classifier revealed that model performance was optimal when using SMOTEENN in conjunction with CoopLearning multi-view feature selection and the DF classifier (Accuracy = 0.9898, multi-class area under the receiver operating characteristic curve = 0.9997).

**Conclusion:**

Collaborative multi-view fusion substantially improved direct multi-class classification across the AD continuum. Integrating clinical and neuroimaging data with Deep Forest may provide a robust and interpretable framework for early auxiliary diagnosis and disease-stage stratification in Alzheimer’s disease.

## 1 Introduction

Alzheimer’s disease (AD) is a neurodegenerative disorder characterized by progressive cognitive dysfunction in older adults. More than 55 million people worldwide are affected by AD (Gauthier et al., 2021), and this number is projected to reach 153 million by 2050 (Nichols et al., 2019). China accounts for approximately one-quarter of the world’s patients with AD aged 60 years or older, creating a growing public-health burden (Lv et al., 2023). AD also affects families and caregivers through psychological and physical strain (Vu et al., 2022). Mild cognitive impairment (MCI) is a clinically important transitional stage between normal cognition (NC) and AD. Individuals with MCI have a higher risk of progressing to AD than cognitively normal individuals, although some may revert to NC (Gao et al., 2018). Early identification of MCI is therefore critical for risk stratification and timely intervention.

Current diagnostic approaches remain limited by subjective interpretation, reduced sensitivity to early pathological change, and difficulty integrating heterogeneous multimodal information (Borchert et al., 2023). Machine learning can support automated interpretation of medical data and improve diagnostic accuracy (Nielsen et al., 2020; Manik, 2023). Multi-view learning is particularly relevant when complementary clinical, biomarker, and neuroimaging variables must be analyzed together. For example, transformer-based multi-view learning has improved mammographic image classification relative to conventional single-view methods (Zhang et al., 2020; Manigrasso et al., 2025). However, multi-view medical data are heterogeneous, and effective modeling requires methods that align complementary information while reducing redundancy and noise.

CoopLearning addresses this challenge by combining feature selection and feature fusion across views. Rather than simply concatenating variables, CoopLearning identifies discriminative information within each view and transfers useful information across views. This cross-view integration can strengthen feature representation and provide a basis for predictive modeling with heterogeneous AD-related data.

Accurate disease modeling also requires classifiers that can capture nonlinear relationships across modalities. Numerous studies have applied machine learning to multi-class AD classification (Richhariya et al., 2020; Shahparian et al., 2021), but conventional approaches often depend on handcrafted features and may have limited capacity to model complex interactions. Deep Forest (DF) (Zhou and Feng, 2019) is an ensemble learning method with an adaptive cascade structure and has shown advantages for direct multi-class classification.

Many AD classification studies have focused on binary discrimination between pairs of diagnostic groups, such as NC versus AD or NC versus MCI (Kim et al., 2021; Nguyen et al., 2023; Zheng et al., 2023). Although binary classifiers can perform well for specific pairwise contrasts, they treat each separation independently and do not directly model the continuum from NC to MCI to AD. This can create inconsistent decision boundaries and limit interpretability when multiple disease states coexist. Direct multi-class classification is therefore better suited to modeling relationships across the full cognitive-aging continuum.

Here, we developed a collaborative multi-view fusion framework combined with DF to classify NC, MCI, and AD directly. By integrating heterogeneous clinical and neuroimaging information from the ADNI database, the framework was designed to evaluate whether CoopLearning-based feature selection and fusion can improve multi-class AD stage classification and provide an interpretable auxiliary model across the AD continuum.

## 2 Materials and methods

### 2.1 Data source

All data were obtained from the ADNI database^1^, a multicenter longitudinal study conducted across multiple clinical research sites. This study comprised 272 subjects (60 AD patients, 140 MCI patients, and 72 NCs) from the ADNI database. Diagnostic labels (NC, MCI, AD) were assigned based on standardized clinical criteria defined by the ADNI protocol, including cognitive assessments and clinical evaluation by expert clinicians. Early and late MCI patients were combined into the MCI group to simplify the analysis. Inclusion criteria were complete baseline data comprising demographic information, biospecimens, scale information, pixel identification, and cranial MRI images. Clinical data were obtained from ADNIMERGE files containing demographic, CSF, and cognitive scale information. MRI data were three-dimensional-magnetization-prepared rapid gradient echo T1-weighted structural MRI (sMRI) images acquired on a 1.5T scanner in digital imaging and communications in medicine format. The specific imaging metric used for classification was gray matter volume. This study is a retrospective predictive modeling study using prospectively collected cohort data.

### 2.2 Data preprocessing

Clinical and imaging data were preprocessed prior to analysis. For the clinical data, according to the literature (Ritchie et al., 2017; Haixiang et al., 2017) and expert recommendations, baseline records were selected, and key demographic and biomarker features were retained. Subjects with available core cerebrospinal fluid biomarkers were included to ensure data reliability. Features with excessive missing values were removed, while remaining missing entries were imputed using mean values to maintain data completeness and consistency. For the imaging data, the baseline T1-weighted sMRI images were preprocessed with MATLAB’s (R2022a) Statistical Parametric Mapping and Voxel-Based Morphometry tools. The images were converted into a unified format, spatially normalized to a common brain template, and non-brain tissues were removed. Subsequently, the images were segmented into major brain tissue types, and gray matter maps were smoothed to reduce noise and enhance data quality. More details are provided in the Supplementary materials.

### 2.3 Feature extraction

The clinical data underwent preprocessing, and the available features were then identified. For the imaging data, we used the REST software (V1.8) to extract features from brain regions using Anatomical Automatic Labeling (AAL), which is a template that divides the brain into 90 regions. The raw gray matter volume values were extracted and subsequently normalized to be used for the analysis.

### 2.4 Feature selection and fusion

#### 2.4.1 Multi-view feature selection and fusion based on CoopLearning

We used the CoopLearning for Multi-view Analysis (Ding et al., 2022) method for feature selection and fusion. CoopLearning is a supervised multi-view learning framework that jointly optimizes prediction accuracy, cross-view agreement, and feature sparsity. Specifically, discriminative features were first selected within each modality to reduce redundancy, followed by cross-modal fusion to integrate complementary information. In this study, 121 variables were divided into five views: demographic information, biospecimen information, relevant scale information, pixel markers, and MRI regional features. Let X_m_ ∈ R^n×p_m_^ denote the feature matrix of the m-th view, where m = 1, M and let y denote the response variable. CoopLearning estimates view-specific coefficient vectors β_m_ by minimizing the following objective function:


$$\underset{\beta_{1},\ldots ,\beta_{M}}{m\,i\,n}\frac{1}{2}\,{\left(y-\sum_{m=1}^{M}X_{m}\,\beta_{m}\right)}^{T}\,\left(y-\sum_{m=1}^{M}X_{m}\,\beta_{m}\right)+$$

$$\frac{\rho }{2}\,\sum_{m=m^{\prime }}{\left(X_{m}\,\beta_{m}-X_{m^{\prime }}\,\beta_{m^{\prime }}\right)}^{T}\,\left(X_{m}\,\beta_{m}-X_{m^{\prime }}\,\beta_{m^{\prime }}\right)+\lambda$$

$$\sum_{m=1}^{M}\left[\left(1-\alpha \right)\,\sum_{j=1}^{p_{m}}\left|\beta_{m\,j}\right|+\frac{\alpha }{2}\,\beta_{m}^{T}\,\beta_{m}\right]$$

The first term measures the prediction error after integrating all views. The second term is the agreement penalty, which encourages the predictions from different views to be consistent. The parameter ρ controls the degree of cooperation among views: when ρ = 0, CoopLearning reduces to early fusion by concatenating features from different views; when ρ = 1, it approximates a late-fusion strategy based on the average of marginal view-specific fits. The third term is an elastic-net regularization term, where λ controls the overall sparsity and α controls the balance between L1 and L2 penalties.

In our implementation, all variables were standardized before model fitting, and missing values were imputed before scaling. CoopLearning was performed with ρ = 0.5 and α = 0.5, which represents an intermediate cooperative setting between early and late fusion while retaining both sparsity and stability. The regularization parameter λ was selected by five-fold cross-validation using cv.multiview, and the model corresponding to lambda.min was used to extract coefficients.

Feature selection was based on the learned coefficients. Features with non-zero coefficients were considered informative and were retained for subsequent modeling. Because all variables were standardized before model fitting, the absolute value of the coefficient was used as an indicator of the relative contribution of each feature within the cooperative multi-view model. For multiclass classification, a feature was retained if it had a non-zero coefficient in at least one class-specific coefficient vector.

For the fusion strategy, CoopLearning generated view-specific linear predictors X_m_β_m_, for each view and optimized them jointly through the agreement penalty, allowing complementary information from different views to be integrated while reducing redundant high-dimensional features. The retained non-zero features from all views were subsequently combined into a fused feature matrix for downstream classification analyses. By encouraging agreement among view-specific predictors, this approach captures aligned and complementary predictive signals across modalities. Compared with traditional early fusion methods, the proposed strategy enhances feature representation, avoids high-dimensional redundancy, and improves the robustness and generalizability of the model. A detailed description is provided in the Supplementary materials.

#### 2.4.2 Other feature selection and fusion methods

Feature selection methods were divided into two types: multi-view least absolute shrinkage and selection operator cross-validation (LassoCV) and single-view LassoCV. Multi-view LassoCV selects features independently from different viewpoints and subsequently combines these features, whereas single-view LassoCV combines features from all viewpoints before selecting them. LassoCV improves on Lasso regression by using L1 regularization to select features, whereas LassoCV automatically determines the optimal regularization parameter through cross-validation.

### 2.5 Category balancing processing

The distribution of categories in real data is often unbalanced, which affects the prediction accuracy of the classifier, necessitating category balancing (Batista et al., 2004). The ADNI dataset used in this study has category imbalance, and data can be balanced by using techniques such as Synthetic Minority Oversampling Technique (SMOTE), SMOTETomek (Chandra and Bedi, 2021), and SMOTE Edited Nearest Neighbors (SMOTEENN) (Chandra and Bedi, 2021). A detailed explanation is provided in the Supplementary materials. To avoid data leakage, class balancing was performed only in the training set. The test set was kept completely untouched and was used only for model evaluation. No synthetic samples or resampling procedures were applied to the test data.

### 2.6 Model development and comparison

To evaluate the performance of different machine learning approaches for Alzheimer’s disease stage prediction, we implemented and compared a range of models, including both ensemble and traditional classifiers. The ensemble methods included Deep Forest (DF) (Zhou and Feng, 2017), Random Forest (RF) (Breiman, 2001), Bagging (Breiman, 1996; Amit and Geman, 1997), Extreme Gradient Boosting (XGBoost) (Chen et al., 2015), and LightGBM (Ke et al., 2017), with DF serving as the primary model due to its adaptive deep cascade structure. Deep Forest was implemented using the CascadeForestClassifier framework. Unlike conventional single-layer tree ensembles, DF builds a cascade structure composed of multiple cascade layers. In each cascade layer, multiple tree-based ensemble estimators are trained, and their class-probability outputs are concatenated with the original input features to form an enhanced feature representation for the next layer. In our implementation, each cascade layer contained two forest estimators, and each estimator consisted of 100 decision trees, and the maximum number of cascade layers was set to 20. The actual number of cascade layers was determined adaptively during training according to the internal out-of-bag validation performance during cascade training. For the tree-level settings, the splitting criterion was Gini impurity, the minimum number of samples required to split an internal node was 2, and the minimum number of samples required at a leaf node was 1. The maximum tree depth was not manually restricted, allowing each tree to expand until the stopping conditions of the tree learner were reached. To avoid unnecessary model growth and overfitting, cascade growth was stopped when the validation performance did not improve by at least 1 × 10^−5^ for two consecutive cascade layers, or when the maximum number of cascade layers was reached. Hyperparameter optimization was performed using a preliminary grid-search strategy with cross-validation. The candidate parameters included the number of forests per cascade layer, maximum tree depth, splitting criterion, minimum samples for node splitting, minimum samples per leaf, binning strategy, backend implementation, and final predictor type. Based on validation performance and model stability, the final DF model was configured with random_state = 42, n_estimators = 2, n_trees = 100, max_layers = 100, max_depth = 2, n_tolerant_rounds = 2, delta = 1 × 10^−5^.

Traditional classifiers comprised Support Vector Machine (SVM) (Chandra and Bedi, 2021), Logistic Regression (LR) (MacKintosh et al., 1990), Decision Tree (DT) (Suthaharan, 2016), and Gaussian Naive Bayes (GNB) (Ontivero-Ortega et al., 2017), implemented using standard multi-class classification strategies. All models were trained and evaluated under consistent experimental settings to ensure fair comparison, and model descriptions are available in the Supplementary materials.

### 2.7 Model evaluation

We divided the dataset into training and testing sets at a ratio of 7:3, and 100 replications were conducted to calculate the arithmetic mean. The model’s performance was evaluated according to both discrimination and calibration metrics. For discrimination, we used accuracy, precision, recall, F1 score, and multi-class area under the receiver operating characteristic curve (MAUC), and the Kappa value was used for calibration metrics.

In addition to the repeated 7:3 train-test split analysis, we performed an additional sensitivity analysis using repeated stratified 10-fold cross-validation under the key setting of CoopLearning-based feature selection and fusion combined with SMOTEENN. The 10-fold cross-validation procedure was repeated 10 times with different random seeds. In each fold, data imputation, scaling, feature selection and fusion, and SMOTEENN-based class balancing were performed using the training fold only, while the test fold was kept untouched and used only for model evaluation. The same cross-validation partitions were used for all nine classifiers to ensure paired model comparison. Model performance was summarized as mean ± standard deviation and 95% confidence interval. Overall differences among classifiers were assessed using the Friedman test, followed by *post hoc* paired Wilcoxon signed-rank tests with Holm-Bonferroni correction when appropriate.

### 2.8 Model implementation

Both multi-view feature selection and fusion were implemented using the “multiview” package in R (version 4.3.2). All other experiments were performed using Python 3.7. The main modules involved are detailed in Supplementary Table 1. To ensure reproducibility, random seeds were fixed before data partitioning, category-balancing, feature selection, and model training. The same random seed was used for NumPy, Python random functions, train-test splitting, SMOTE-based resampling procedures, and classifiers with stochastic components. All experiments were repeated 100 times under the same experimental protocol, and the arithmetic mean of each evaluation metric was reported. The main hyperparameters of all classifiers were explicitly recorded and are provided in Supplementary Table 1.

## 3 Results

### 3.1 Basic information about the research subjects

The descriptive statistics and demographic data are presented in Table 1.

**TABLE 1:** Descriptive statistics and demographic information of the study subjects.

Feature/variable	AD	MCI	NC
Numbers	60	140	72
Age	76.16 ± 6.56	74.62 ± 7.48	77.00 ± 5.75
Women	31 (51.67)	93 (66.43)	41 (56.94)
Men	29 (48.33)	47 (33.57)	31 (43.06)
Years of education	14.42 ± 2.83	15.69 ± 2.97	16.08 ± 2.51
Married	51 (85.00)	113 (80.71)	50 (69.44)
Divorce	2 (3.33)	8 (5.71)	6 (8.33)
Widower	5 (8.33)	17 (12.14)	14 (19.44)
Unmarried	2 (3.33)	2 (1.43)	2 (2.78)
Carried APOE4	42 (70.00)	73 (52.14)	22 (30.56)
Uncarried APOE4	18 (30.00)	67 (47.86)	50 (69.44)

Values represent number (%) or mean ± standard deviation. APOE4, a genetic risk allele of the APOE gene associated with increased susceptibility to Alzheimer’s disease.

### 3.2 Feature extraction results

#### 3.2.1 Clinical data feature extraction results

The clinical data were extracted after data preprocessing, and the total number of features extracted was 31, in four views, as shown in Supplementary Table 2.

#### 3.2.2 Imaging data feature extraction results

90 brain regions were extracted using the AAL template.

### 3.3 Feature selection results

#### 3.3.1 CoopLearning feature selection results

The CoopLearning feature selection process filtered out 20 variables, and the sub-view presentation results are displayed in Table 2.

**TABLE 2:** CoopLearning feature selection results.

View name	Feature number	Variable
Personal information view	4	AGE, PTGENDER, PTEDUCAT, APOE4
Biological sample information view	2	ABETA_bl, PTAU_bl
View of the related scale information	6	CDRSB_bl, ADASQ4_bl, MMSE_bl, RAVLT_perc_forgetting_bl, LDELTOTAL_BL, FAQ_bl
Pixel identification view	3	Ventricles_bl, WholeBrain_bl, Fusiform_bl
Characteristic view of the MRI region	5	Precuneus (left and right), bean-like pallidum (right), thalamus (left), superior temporal gyrus cortex (right)

#### 3.3.2 LassoCV multi-view feature selection results

The LassoCV multi-view feature selection process identified 20 variables, and the view-specific results are displayed in Supplementary Table 3.

#### 3.3.3 Overall single-view feature selection results

The overall single-view feature selection process identified 20 variables, and the view-specific results are displayed in Supplementary Table 4.

### 3.4 Model classification results

#### 3.4.1 CoopLearning feature selection and fusion classification results

Table 3 shows the performance of the classifiers improved after data preprocessing with SMOTE, SMOTETomek, and SMOTEENN. In most cases, the dataset processed using CoopLearning and SMOTEENN yielded the best classifier performance. Accuracy, F1 score, recall, and Kappa values were higher following SMOTEENN preprocessing. Notably, the performance of the DF integrated classifier showed a significant improvement, with accuracy increasing from 0.9305 to 0.9898 and the Kappa value improving from 0.8872 to 0.9840 after conducting SMOTEENN category balancing on the original data.

**TABLE 3:** CoopLearning results using classifiers.

Category balance processing		Classifier	Accuracy	F1	Precision	Recall	MAUC	Kappa
Initial data	Integrated classifier	DF	**0.9305**	**0.9235**	**0.9325**	**0.9230**	**0.9879**	**0.8872**
		RF	0.9256	0.9182	0.9302	0.9113	0.9852	0.8783
		Bagging	0.9037	0.8954	0.9259	0.8770	0.9653	0.8396
		XGBoost	0.9244	0.9191	0.9270	0.9147	0.9733	0.8766
		LightGBM	0.9232	0.9174	0.9303	0.910	0.9837	0.8741
	Traditional classifier	SVM	0.8659	0.8548	0.9064	0.8277	0.9762	0.7727
		GNB	0.9037	0.9019	0.8984	0.9146	0.9768	0.8471
		Logistic	0.9134	0.9084	0.9367	0.8903	0.9843	0.8556
		DT	0.9183	0.9131	0.9237	0.9057	0.9427	0.8663
SMOTE	Integrated classifier	DF	**0.9603**	**0.9602**	**0.9618**	**0.9603**	**0.9951**	**0.9405**
		RF	0.9557	0.9557	0.9572	0.9557	0.9930	0.9335
		Bagging	0.9365	0.9358	0.9372	0.9365	0.9827	0.9048
		XGBoost	0.9451	0.9451	0.9476	0.9451	0.9877	0.9177
		LightGBM	0.9464	0.9465	0.9485	0.9464	0.9915	0.9196
	Traditional classifier	SVM	0.9259	0.9254	0.9270	0.9259	0.9892	0.8889
		GNB	0.9206	0.9196	0.9214	0.9206	0.9858	0.8810
		Logistic	0.9325	0.9319	0.9335	0.9325	0.9914	0.8988
		DT	0.9160	0.9164	0.9198	0.9160	0.9564	0.8740
SMOTETomek	Integrated classifier	DF	**0.9668**	**0.9667**	**0.9679**	**0.9668**	**0.9954**	**0.9502**
		RF	0.9517	0.9516	0.9532	0.9517	0.9928	0.9275
		Bagging	0.9307	0.9301	0.9311	0.9307	0.9788	0.8961
		XGBoost	0.9430	0.9430	0.9450	0.9430	0.9884	0.9145
		LightGBM	0.9459	0.9459	0.9477	0.9459	0.9928	0.9188
	Traditional classifier	SVM	0.9257	0.9253	0.9266	0.9257	0.9893	0.8885
		GNB	0.9206	0.9208	0.9235	0.9206	0.9854	0.8810
		Logistic	0.9300	0.9293	0.9308	0.9300	0.9909	0.8950
		DT	0.9113	0.9112	0.9163	0.9113	0.9529	0.8669
SMOTEENN	Integrated classifier	DF	**0.9898**	**0.9872**	**0.9883**	**0.9865**	**0.9997**	**0.9840**
		RF	0.9754	0.9688	0.9744	0.9649	0.9966	0.9615
		Bagging	0.9649	0.9542	0.9649	0.9481	0.9900	0.9447
		XGBoost	0.9719	0.9637	0.9718	0.9591	0.9902	0.9559
		LightGBM	0.9711	0.9629	0.9689	0.9598	0.9939	0.9546
	Traditional classifier	SVM	0.9526	0.9372	0.9545	0.9276	0.9970	0.9251
		GNB	0.9632	0.9544	0.9542	0.9561	0.9949	0.9426
		Logistic	0.9439	0.9240	0.9494	0.9115	0.9965	0.9109
		DT	0.9570	0.9456	0.9525	0.9437	0.9706	0.9327

Bold indicates the best value for each evaluation metric across the different models.

#### 3.4.2 LassoCV multi-view feature selection classification results

Supplementary Table 5 shows when compared with the original data, data preprocessed with SMOTE, SMOTETomek, and SMOTEENN had better classifier performance (with the exception of the SVM results post-SMOTEENN processing). Among the four category balancing strategies, the LassoCV multi-view feature selection yielded the best classifier performance for datasets processed with SMOTETomek after early fusion. Specifically, the DF classifier achieved the highest performance with an accuracy of 0.9765 and a Kappa value of 0.9646.

#### 3.4.3 Overall single view feature selection classification results

Supplementary Table 6 shows when all features were combined into a single view, overall feature selection resulted in relatively poor performance for all classifiers without the category-balancing process. DF performed optimally under the SMOTEENN balancing technique, achieving an accuracy of 0.9032 and a Kappa value of 0.8242. However, it did not outperform datasets processed by SMOTE or SMOTETomek in terms of precision, recall, MAUC, or F1 score. The DF classifier under the SMOTEENN category-balancing approach generated the highest accuracy and Kappa values. This classifier was the top performer among the models using various balancing techniques.

#### 3.4.4 Classification performance of Deep Forest under CoopLearning after SMOTEENN-based class balancing

Figure 1 shows that the model accurately distinguishes AD from NC, with no AD cases misclassified as NC and no NC cases misclassified as AD. However, misclassification remains in the transitional MCI stage: 0.02 of NC cases were classified as MCI, 0.16 of MCI cases as NC, 0.41 of MCI cases as AD, and 0.38 of AD cases as MCI.

**FIGURE 1:**
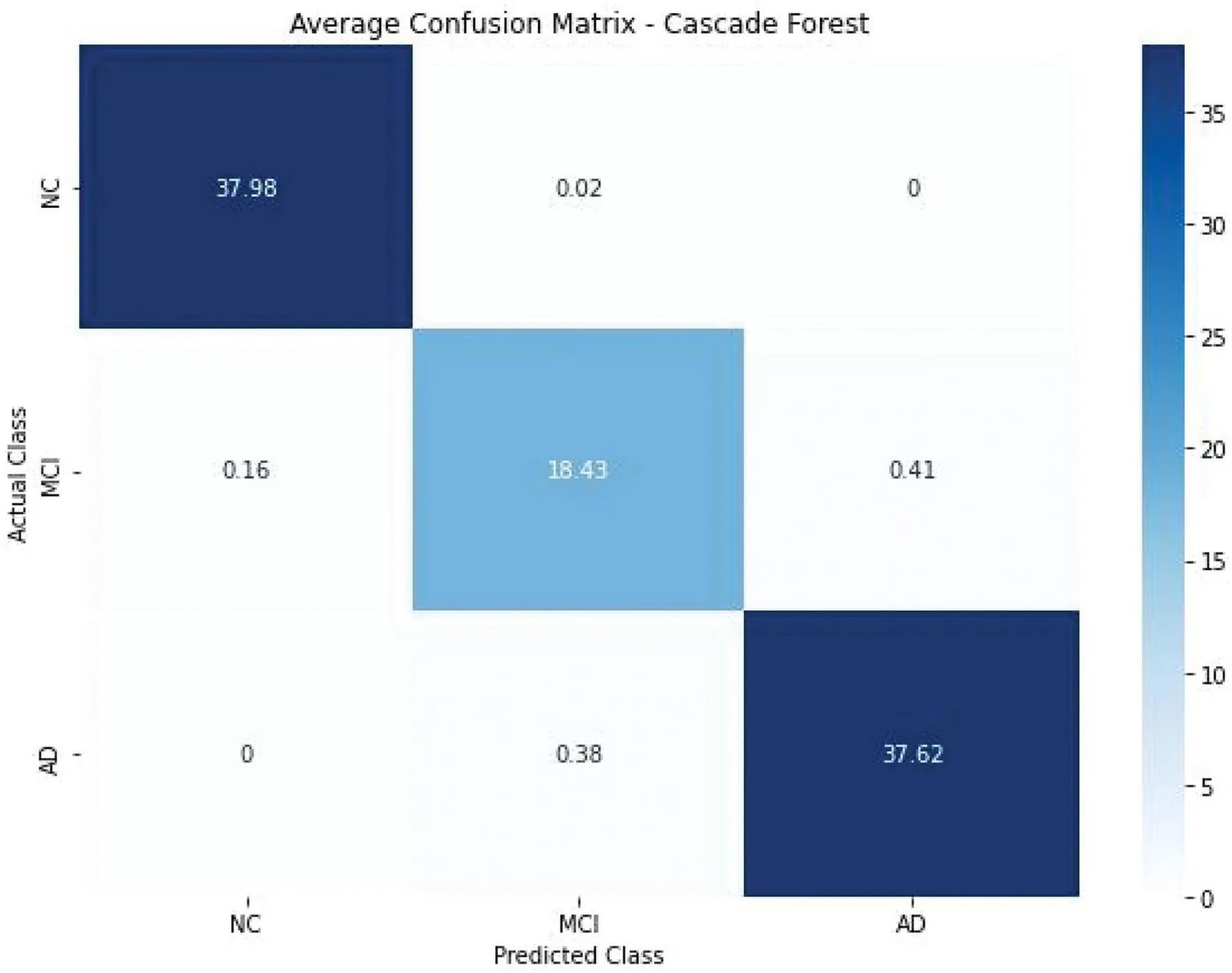
Average confusion matrix of DF classification results under CoopLearning after SMOTEENN processing.

#### 3.4.5 Synthesis of model results

The best-performing models across category-balancing methods for CoopLearning, LassoCV multi-view feature selection with early fusion, and LassoCV overall single-view feature selection are summarized in Table 4. Multi-view feature selection used the dataset information more effectively than overall single-view feature selection and improved model performance. Although DF performance varied across feature-selection methods and fusion strategies, it performed well in multi-view settings. Overall, ensemble learning approaches were generally superior to traditional machine learning models when applied to the same dataset. The best classification performance was achieved by the DF classifier combined with SMOTEENN under the CoopLearning method. For LassoCV multi-view feature selection with early fusion, the best results were obtained with SMOTETomek. These findings indicate that selecting a category-balancing method according to data-distribution characteristics can improve model performance.

**TABLE 4:** Optimal classifiers of three methods with different category-balancing methods.

Optimal classifiers of methods with different class leveling methods	Category balance processing	Classifier	Accuracy	F1	Precision	Recall	MAUC	Kappa
CoopLearning	Initial data	DF	0.9305	0.9235	0.9325	0.9230	0.9879	0.8872
	SMOTE	DF	0.9603	0.9602	0.9618	0.9603	0.9951	0.9405
	SMOTETomek	DF	0.9668	0.9667	0.9679	0.9668	0.9954	0.9502
	SMOTEENN	DF	**0.9898**	**0.9872**	**0.9883**	**0.9865**	**0.9997**	**0.984**
Multi-view LassoCV combined with early fusion	Initial data	DF	0.9276	0.9193	0.9365	0.9098	0.9862	0.8809
	SMOTE	DF	0.9476	0.9474	0.9484	0.9476	0.9943	0.9214
	SMOTETomek	DF	**0.9765**	**0.9759**	0.9773	**0.9754**	**0.9966**	**0.9646**
	SMOTEENN	DF	0.9714	0.9504	**0.9792**	0.9333	0.9960	0.9512
Overall single view feature selection	Initial data	DF	0.6707	0.6369	0.6876	0.6236	0.8387	0.4383
	SMOTE	DF	0.7841	0.7790	0.7874	0.7841	**0.9280**	0.6762
	SMOTETomek	DF	0.8155	**0.8116**	**0.8212**	**0.8131**	0.9243	0.7231
	SMOTEENN	DF	**0.9032**	0.6664	0.6735	0.6844	0.8788	**0.8242**

Bold indicates the best value for each feature selection method. DF, Deep Forest.

#### 3.4.6 Sensitivity analysis and statistical comparison of classifiers

To further address the possibility that the high performance was driven by a favorable train-test split, we performed a sensitivity analysis using 10 repeats of stratified 10-fold cross-validation under the key setting of CoopLearning-based feature selection and fusion combined with SMOTEENN. All nine classifiers were evaluated using the same cross-validation partitions, and the results are shown in Supplementary Table 7. Compared with the repeated 7:3 train-test split analysis, the repeated cross-validation produced more conservative performance estimates. Nevertheless, the DF classifier remained the best-performing model across all evaluation metrics, with an Accuracy of 0.915 ± 0.049, F1-score of 0.913 ± 0.049, Precision of 0.912 ± 0.047, Recall of 0.924 ± 0.048, MAUC of 0.982 ± 0.018, and Kappa of 0.864 ± 0.077. The corresponding 95% CIs were 0.905–0.924 for Accuracy, 0.903–0.922 for F1-score, 0.903–0.921 for Precision, 0.915–0.934 for Recall, 0.978–0.986 for MAUC, and 0.849–0.879 for Kappa.

The Friedman test showed significant overall differences among the nine classifiers for all six evaluation metrics, including Accuracy, F1-score, Precision, Recall, MAUC, and Kappa, with all *P*-values < 0.001. *Post hoc* paired Wilcoxon signed-rank tests with Holm-Bonferroni correction showed that the DF classifier achieved significantly higher Accuracy than Bagging, DT, GNB, LightGBM, Logistic Regression, and SVM. However, the differences between DF and RF or XGBoost did not reach statistical significance after correction. These results suggest that the superior performance of DF was generally stable across repeated cross-validation partitions, although the cross-validation estimates were more conservative than those obtained from the repeated 7:3 train-test split analysis.

## 4 Discussion

This study developed a CoopLearning-based multi-view framework combined with DF for direct classification of NC, MCI, and AD. By integrating clinical and MRI-derived information, the framework improved AD stage classification after feature selection, feature fusion, and category balancing. The strongest performance was obtained when CoopLearning was combined with SMOTEENN and the DF classifier, supporting the value of collaborative multi-view fusion for auxiliary AD-stage stratification.

The selected features also support the biological plausibility and clinical interpretability of the framework. MRI-selected regions, including the bilateral precuneus, right lentiform pallidum, left thalamus, and right superior temporal gyrus, are closely related to AD-associated neurodegeneration. The precuneus (Raichle, 2015) is a key hub of the default mode network and is involved in memory decline and early network disruption. The thalamus (Vercruysse et al., 2018) supports cortical-subcortical communication and may affect memory, attention, and global cognition, whereas the superior temporal gyrus (Li et al., 2021) is associated with language and semantic memory. Pallidal abnormalities (Zhang et al., 2021) may reflect subcortical circuit involvement in cognitive, emotional, and motor regulation.

Non-regional features further strengthened the clinical meaning of the model. Age, sex, education, and APOE4 status represent demographic and genetic risk factors; ABETA_bl and PTAU_bl (Goate et al., 1991) reflect amyloid and tau pathology; cognitive and functional scales such as CDRSB (Tzeng et al., 2022), ADASQ4 (Rosen et al., 1984), MMSE (Folstein et al., 1975), RAVLT (Moradi et al., 2017), and FAQ capture cognitive decline, memory impairment, and reduced daily functioning; and ventricular, whole-brain, and fusiform volumes reflect structural atrophy. Therefore, model predictions can be interpreted as evidence derived from multiple clinically meaningful domains rather than as purely black-box outputs. Clinical translation of AI models depends on interpretability and trust. Islam et al. (2025) noted that explainable AI methods, including SHAP, and knowledge-graph-based representation, can improve the transparency of AI-assisted healthcare recommendations. In this study, interpretability is partly supported by view-specific feature selection and AAL-defined MRI regional features, as the selected variables can be linked to meaningful demographic, biochemical, cognitive, and neuroanatomical domains. However, formal *post hoc* explainability methods were not included. Future work should incorporate SHAP-based attribution, view-level contribution analysis, and knowledge-guided interpretation to explain individual NC, MCI, and AD predictions.

Direct multi-class classification addresses an important limitation of pairwise AD classification. Many previous studies have distinguished NC from AD or NC from MCI (Park et al., 2023), but separate binary classifiers do not fully capture the continuum of cognitive decline. Combining binary decisions after model training can also introduce inconsistent boundaries across disease states. A unified multi-class model jointly represents NC, MCI, and AD, which may better reflect the transitions from normal cognition through MCI to AD and provide a more coherent framework for stage stratification.

Multi-view fusion is particularly useful for AD research because clinically relevant information is distributed across data types. Neuroimaging biomarkers can show the extent of lesions in patients with AD and help detect disease stages (Zhang et al., 2021), but imaging acquisition is costly and sample sizes are often limited. Data augmentation methods have therefore been used to increase the amount of data available for modeling (Goceri, 2023). In the present study, the AAL template was used to extract 90 MRI brain-region features, and feature selection allowed the classifier to focus on brain regions related to AD progression. Because each feature corresponded to an AAL template region, the model retained explanatory value while reducing the amount of data required for training.

The CoopLearning-based strategy provided a data-level approach to multi-view feature selection and fusion. By using information from different views, CoopLearning can support disease classification and prediction in heterogeneous biomedical datasets. Ding et al. (2023) proposed a semi-supervised co-learning framework that integrates multimodal biomedical data, further supporting the relevance of cooperative learning strategies. In our comparison, LassoCV was applied to both multi-view feature selection and overall single-view feature selection. The overall single-view method concatenated all view data before feature selection, whereas multi-view approaches retained view-specific information before integration. This difference helps explain why multi-view feature selection improved prediction accuracy.

While more complex deep neural network architectures, including those integrating optimization algorithms such as the Gray Wolf Optimizer, have demonstrated effectiveness in other tumor identification tasks (Khair et al., 2025), we selected the DF classifier. As an ensemble method, DF is particularly suited for biomedical datasets characterized by limited sample sizes and high-dimensional features, as it requires minimal hyperparameter tuning and achieves robust performance without intensive computational demands. The performance of DF is also relevant to the small-sample setting of aging and neurodegenerative disease research. The ADNI dataset, like many longitudinal aging cohorts, is constrained by the cost of multimodal data collection and long-term follow-up in older populations. DF can perform well with small samples and default parameters, reducing the need for extensive hyperparameter tuning. Cascade forest regression, an improved model based on DF, has been used for automatic blood pressure estimation and improved accuracy even with small samples and default parameters (Zhang et al., 2023). In another comparison of classifiers for drug-target interaction prediction, DF achieved the highest performance across several datasets (Lian et al., 2023). These findings are consistent with the strong performance of DF in the present multi-view AD classification setting. Several points should be considered when interpreting these findings. This study used ADNI data and evaluated a retrospective predictive modeling workflow. The results support the potential of CoopLearning-based multi-view fusion and DF for auxiliary AD-stage stratification, but the framework should be interpreted within the dataset, feature set, and evaluation strategy used here. Additional validation would be needed before broader clinical use.

The study has several strengths. First, imaging data were integrated with clinical information to construct a multi-view dataset, reducing the limitations of single-view data and improving model stability. Second, CoopLearning enabled both view-specific feature selection and data-level cross-view fusion, preserving complementary information across modalities. Third, the direct multi-class classification strategy jointly modeled NC, MCI, and AD, providing a more coherent and efficient framework for AD stage stratification and auxiliary diagnosis.

This study has several limitations. First, the analysis was based only on the ADNI database, and external validation in independent cohorts was not performed. Although the CoopLearning-SMOTEENN-DF model achieved high classification performance, the possibility of overfitting should be considered because of the relatively small sample size. The repeated stratified cross-validation, confidence intervals, and statistical comparisons provide additional evidence of model stability, but they cannot replace external validation. Future studies should validate the framework in independent multicenter cohorts before clinical application. Second, incorporating genomic, proteomic, metabolomic, or other molecular biomarkers may further improve AD stage stratification and support precision neurology (Manik et al., 2025). Finally, interpretability was mainly based on selected features and view-specific information, which may not fully explain nonlinear interactions learned by DF. Future work should apply explainability methods such as SHAP, counterfactual explanations, and individual-level decision reports to improve clinical trust and real-world adoption.

In summary, we constructed a CoopLearning-based multi-view fusion framework combined with a DF classifier for auxiliary multi-class AD stage classification. The framework achieved high internal performance within the ADNI cohort, suggesting its potential value for early AD-stage stratification. Nevertheless, external validation in independent multicenter cohorts is required before the model can be generalized to broader populations or considered for real-world clinical application.

## Data Availability

The datasets presented in this study were obtained from the Alzheimer’s Disease Neuroimaging Initiative (ADNI) database (https://adni.loni.usc.edu/), and the study data are available from the corresponding author upon reasonable request.

## Footnotes

1
https://adni.loni.usc.edu/
