# Supplementary material for: A collaborative multi-view fusion framework with Deep Forest for multi-class diagnosis of Alzheimer’s disease
Source: Front Aging Neurosci. 2026 Sep 24;18:1888587. doi: 10.3389/fnagi.2026.1888587 (PMC13646108; doi:10.3389/fnagi.2026.1888587)
Supplement: Supplementary file 1 [file Table_1.docx]

**Supplement**

**2.3 Data Preprocessing**

**2.3.1 Clinical Data Preprocessing**

The baseline data of the ADNIMERGE file with ORIGPROT as ADNI1, VISCODE were screened. After retaining Personal Information View features (AGE, PTGENDER, PTEDUCAT, PTMARRY, and APOE4), other features with the _bl suffix (baseline data) only were retained. For additional screening, according to the literature^[17-18]^ and expert recommendations, subjects with records for ABETA_bl, TAU_bl, and PTAU_bl (three CSF biomarker indicators) were selected. Features with missing rates greater than 0.5 were removed, and means were used to fill in the remaining missing variables (with a maximum missing rate of 0.204). Specifically, for each variable that had missing values, we calculated the average of its existing non-missing values. This average value was used for all missing values for that variable. Only variables with ≤ 20.4% missing values were treated in this way.

**2.3.2 Image Data Preprocessing**

The baseline T1-weighted sMRI images were preprocessed with MATLAB’s (R2022a) Statistical Parametric Mapping and Voxel-Based Morphometrytools. The sMRI images were converted from DICOM format to Nifti format and normalized to Montreal Neurological Institute space. The skull and cerebellum were removed from the images, followed by segmentation into white matter, gray matter, and CSF. The gray matter images were smoothed using an 8-mm full width at half maximum kernel to reduce noise.

**2.5 Feature Selection and Fusion**

**2.5.1 Multi-view Feature Selection and Fusion Based on CoopLearning**

We used the CoopLearning for Multi-view Analysis^[19]^ method for feature selection and fusion. CoopLearning emphasizes dynamic and hierarchical collaboration: first, the feature selection mechanism is used to filter modal features to retain highly discriminative features and suppress redundant information; then, in the feature fusion stage, cross-modal correlations are constructed in the feature space by adaptive weighting, kernel methods, or deep neural networks to realize the nonlinear integration of complementary information. This two-way synergy of “selection-fusion” not only avoids the dimensionality disaster of early fusion but also breaks through the expressive limitations of a single data source and significantly improves the robustness and generalizability of the model by building semantic bridges between features. Where traditional early fusion combines features from each view after feature selection, CoopLearning exploits interactions between views to improve model performance.

**2.6 Category Balancing Processing**

In SMOTETomek^[27]^, SMOTETomek links eliminate overlapping samples by removing pairs of nearest neighbors of different categories. In SMOTEENN^[27]^, SMOTE functions in the same way, while edited nearest neighbors decide whether to remove a sample by determining the k-nearest neighbor (KNN) categories of each sample.

**2.7 Model Development and Comparison**

**Integrated Classifiers**

(1) **DF**

DF^[21]^ is an alternative to deep neural networks (DNNs) and is based on DTs, as proposed by Zhou *et al.* DF intermediate data can be output and layers can be added adaptively to increase complexity.

(2) **RF**

RF^[22]^ is an integrated learning method based on DTs. It is a special form of the Bagging method inspired by Breiman^[23]^.

(3) **Bagging**

Bagging^[24]^ is an approach that integrates learning algorithms by combining multiple models to enhance the stability and accuracy of a single model.

(4) **XGBoost**

XGBoost^[25]^ is an integrated learning method that builds upon Gradient Boosting DTs (GBDTs), incorporating optimizations and enhancements to GBDT. XGBoost possesses strong model generalizability while ensuring prediction accuracy.

(5) **LightGBM**

LightGBM^[26]^ is an integrated learning method based on GBDT. LightGBM enhances training speed without compromising accuracy by using gradient-based one-side sampling and exclusive feature binding algorithms.

**Traditional Classifiers**

(1) **SVM**

SVM^[27]^ was originally designed as a supervised learning method for binary classification problems to distinguish two categories by identifying a hyperplane that maximizes the margin between them. We applied an SVM for the multiclassification task using the one-to-one (OvO) approach.

(2) **LR**

LR^[28]^, known as the log odds model, is a widely used statistical method. It is an algorithm that classifies samples by linearly stacking them and applying a sigmoid function. We used LR for the triple classification task using the one-versus-all approach.

(3) **DTs**

DTs^[29]^recursively select the most effective features and partition the training data to optimally classify each subset. The construction of a DT typically involves three steps: feature selection, DT generation, and DT pruning.

(4) **GNB**

GNB^[30]^ is a plain Bayesian classifier that can be used for direct multiclassification tasks. A simple Bayesian classifier is termed ‘simple’ because it assumes feature independence, whereby each feature independently influences the classification.

Supplement Table S1. Python libraries and modules used by the model

| Library | Module | Function | Parameter details |
| --- | --- | --- | --- |
| sklearn | impute | SimpleImputer | strategy='mean' |
| sklearn | linear_model | LassoCV | cv=5, random_state=42, max_iter=10000 |
| imblearn | over_sampling | SMOTE | random_state=21 |
| imblearn | combine | SMOTETomek, SMOTEENN | random_state=21 |
| deepforest |  | CascadeForestClassifier | n_jobs=-1, random_state=42, max_layers=100, n_estimators=2, max_depth=2, n_tolerant_rounds = 2, verbose=0 |
| sklearn | ensemble | RandomForestClassifier, | random_state=42, n_estimators=6 |
| sklearn | ensemble | BaggingClassifier | base_estimator=SVC(), random_state=42 |
| sklearn | svm | SVC | random_state=42, random_state=42, probability=True, tol=0.01, degree=1, C=0.2 |
| sklearn | linear_model | LogisticRegression | random_state=42 |
| sklearn | tree | DecisionTreeClassifier | random_state=42, max_leaf_nodes=3 |
| sklearn | naive_bayes | GaussianNB | var_smoothing=1e-2 |
| xgboost |  | XGBClassifier | random_state=42, n_estimators=2, max_depth=2 |
| lightgbm |  | LGBMClassifier | random_state=42, n_estimators=5, verbose=-1 |
| sklearn | metrics | confusion_matrix, roc_auc_score, accuracy_score, f1_score  precision_score, recall_score, cohen_kappa_score, matthews_corrcoef |  |

Supplement Table S2. Features extracted from clinical data after data preprocessing

| View name | Feature number | Variable |
| --- | --- | --- |
| Personal Information View | 5 | AGE, PTGENDER, PTEDUCAT, PTMARRY, APOE4 |
| Biological Sample Information View | 3 | ABETA_bl, TAU_bl, PTAU_bl |
| View of the related scale information | 16 | CDRSB_bl, ADAS11_bl, ADASQ4_bl, ADAS13_bl, MMSE_bl, RAVLT_immediate_bl, RAVLT_learning_bl, RAVLT_forgetting_bl, RAVLT_perc_forgetting_bl, LDELTOTAL_BL, DIGITSCOR_bl, TRABSCOR_bl, FAQ_bl, mPACCdigit_bl, mPACCtrailsB_bl, IMAGEUID_bl |
| Pixel identification view | 7 | Ventricles_bl, Hippocampus_bl, WholeBrain_bl, Entorhinal_bl, Fusiform_bl, MidTemp_bl, ICV_bl |

Supplement Table S3. LassoCV multi-view feature selection results

| View name | Feature number | Variable |
| --- | --- | --- |
| Personal Information View | 3 | PTEDUCAT, PTMARRY, APOE4 |
| Biological Sample Information View | 1 | TAU_bl |
| View of the related scale information | 7 | CDRSB_bl, ADAS11_bl RAVLT_immediate_bl, LDELTOTAL_BL, FAQ_bl, mPACCdigit_bl, mPACCtrailsB_bl |
| Pixel identification view | 6 | Ventricles_bl, Hippocampus_bl, Entorhinal_bl, Fusiform_bl, MidTemp_bl, ICV_bl |
| Characteristic view of the MRI region | 3 | Middle occipital gyrus (left and right), paracentral leaflet (left) |

Supplement Table S4. Overall single view feature selection results

| View name | Feature number | Variable |
| --- | --- | --- |
| Personal Information View | 0 | / |
| Biological Sample Information View | 2 | ABETA_bl, PTAU_bl |
| View of the related scale information | 3 | RAVLT_perc_forgetting_bl, TRABSCOR_bl, IMAGEUID_bl |
| Pixel identification view | 6 | Ventricles_bl, Hippocampus_bl, WholeBrain_bl, Entorhinal_bl, MidTemp_bl, ICV_bl |
| Characteristic view of the MRI region | 9 | Anterior central gyrus (left and right), parahippocampal gyrus (left), middle occipital gyrus (left and right), fusiform gyrus (left and right) |

Supplement Table S5. Results of the LassoCV multi-view feature selection after early fusion using classifiers

| Category balance processing | | Classifier | Accuracy | F1 | Precision | Recall | MAUC | Kappa |
| --- | --- | --- | --- | --- | --- | --- | --- | --- |
| initial data | integrated classifier | DF | **0.9276** | **0.9193** | **0.9365** | 0.9098 | **0.9862** | **0.8809** |
|  |  | RF | 0.9085 | 0.8958 | 0.9213 | 0.8831 | 0.9745 | 0.8482 |
|  |  | Bagging | 0.9055 | 0.8965 | 0.9299 | 0.8776 | 0.9631 | 0.8423 |
|  |  | XGBoost | 0.9169 | 0.9089 | 0.9234 | 0.9006 | 0.9689 | 0.8635 |
|  |  | LightGBM | 0.9200 | 0.9131 | 0.9256 | 0.9045 | 0.9723 | 0.8687 |
|  | traditional classifier | SVM | 0.9024 | 0.8967 | 0.9263 | 0.8789 | 0.9818 | 0.8369 |
|  |  | GNB | 0.8125 | 0.7773 | 0.9016 | 0.7400 | 0.9706 | 0.6698 |
|  |  | Logistic | 0.8994 | 0.8975 | 0.8944 | **0.9099** | 0.9752 | 0.8404 |
|  |  | DT | 0.9093 | 0.9024 | 0.9369 | 0.8819 | 0.9830 | 0.8479 |
| SMOTE | integrated classifier | DF | **0.9476** | **0.9474** | **0.9484** | **0.9476** | **0.9943** | **0.9214** |
|  |  | RF | 0.9425 | 0.9425 | 0.9455 | 0.9425 | 0.9901 | 0.9137 |
|  |  | Bagging | 0.9380 | 0.9377 | 0.9388 | 0.9380 | 0.9814 | 0.9070 |
|  |  | XGBoost | 0.9211 | 0.9213 | 0.9247 | 0.9211 | 0.9791 | 0.8817 |
|  |  | LightGBM | 0.9266 | 0.9270 | 0.9310 | 0.9266 | 0.9819 | 0.8899 |
|  | traditional classifier | SVM | 0.9256 | 0.9259 | 0.9293 | 0.9256 | 0.9866 | 0.8884 |
|  |  | GNB | 0.9142 | 0.9140 | 0.9156 | 0.9142 | 0.9861 | 0.8713 |
|  |  | Logistic | 0.9182 | 0.9185 | 0.9210 | 0.9182 | 0.9851 | 0.8772 |
|  |  | DT | 0.9143 | 0.9147 | 0.9231 | 0.9143 | 0.9546 | 0.8714 |
| SMOTETomek | integrated classifier | DF | **0.9765** | **0.9759** | **0.9773** | **0.9754** | **0.9966** | **0.9646** |
|  |  | RF | 0.9706 | 0.9701 | 0.9706 | 0.9712 | 0.9950 | 0.9559 |
|  |  | Bagging | 0.9510 | 0.9495 | 0.9508 | 0.9498 | 0.9912 | 0.9263 |
|  |  | XGBoost | 0.9569 | 0.9563 | 0.9582 | 0.9580 | 0.9942 | 0.9354 |
|  |  | LightGBM | 0.9588 | 0.9582 | 0.9592 | 0.9599 | 0.9936 | 0.9382 |
|  | traditional classifier | SVM | 0.9412 | 0.9392 | 0.9409 | 0.9394 | 0.9939 | 0.9115 |
|  |  | GNB | 0.9373 | 0.9348 | 0.9382 | 0.9341 | 0.9915 | 0.9055 |
|  |  | Logistic | 0.9333 | 0.9310 | 0.9335 | 0.9310 | 0.9925 | 0.8997 |
|  |  | DT | 0.9373 | 0.9364 | 0.9398 | 0.9386 | 0.9694 | 0.9060 |
| SMOTEENN | integrated classifier | DF | **0.9714** | **0.9504** | **0.9792** | 0.9333 | **0.9960** | **0.9512** |
|  |  | RF | 0.9657 | 0.9330 | 0.9736 | 0.9200 | 0.9907 | 0.9412 |
|  |  | Bagging | 0.9543 | 0.9260 | 0.9417 | 0.9167 | 0.9917 | 0.9223 |
|  |  | XGBoost | 0.9600 | 0.9436 | 0.9616 | **0.9378** | 0.9755 | 0.9314 |
|  |  | LightGBM | 0.9600 | 0.9387 | 0.9771 | 0.9222 | 0.9909 | 0.9304 |
|  | traditional classifier | SVM | 0.9086 | 0.8032 | 0.9367 | 0.7867 | 0.9909 | 0.8394 |
|  |  | GNB | 0.9314 | 0.8614 | 0.9488 | 0.8400 | 0.9713 | 0.8814 |
|  |  | Logistic | 0.9314 | 0.8579 | 0.9472 | 0.8400 | 0.9795 | 0.8812 |
|  |  | DT | 0.9371 | 0.9003 | 0.9472 | 0.8919 | 0.9517 | 0.8947 |

Note: Bold indicates the best value for each evaluation metric across the different models.

Supplement Table S6. Results of classifiers after overall single-view feature selection

| Category balance processing | | Classifier | Accuracy | F1 | Precision | Recall | MAUC | Kappa |
| --- | --- | --- | --- | --- | --- | --- | --- | --- |
| Initial data | integrated classifier | DF | **0.6707** | **0.6369** | 0.6876 | **0.6236** | **0.8387** | **0.4383** |
|  |  | RF | **0.6707** | 0.6268 | 0.6965 | 0.608 | 0.8342 | 0.4293 |
|  |  | Bagging | 0.6439 | 0.6090 | 0.6902 | 0.5835 | 0.801 | 0.3780 |
|  |  | XGBoost | 0.6390 | 0.6228 | 0.6363 | 0.6148 | 0.8071 | 0.4027 |
|  |  | LightGBM | 0.6439 | 0.6314 | 0.6495 | 0.6215 | 0.8177 | 0.4098 |
|  | traditional classifier | SVM | 0.6341 | 0.5927 | 0.6801 | 0.5724 | 0.8269 | 0.3623 |
|  |  | GNB | 0.4927 | 0.4938 | 0.5585 | 0.6101 | 0.8036 | 0.2932 |
|  |  | Logistic | 0.6585 | 0.6169 | **0.7004** | 0.5905 | 0.8345 | 0.4003 |
|  |  | DT | 0.5902 | 0.5837 | 0.5849 | 0.5866 | 0.6794 | 0.3400 |
| SMOTE | integrated classifier | DF | **0.7841** | **0.7790** | **0.7874** | **0.7841** | **0.9280** | **0.6762** |
|  |  | RF | 0.7690 | 0.7574 | 0.7668 | 0.764 | 0.9185 | 0.6526 |
|  |  | Bagging | 0.7483 | 0.7433 | 0.7483 | 0.7449 | 0.8714 | 0.6221 |
|  |  | XGBoost | 0.7586 | 0.7462 | 0.7511 | 0.7534 | 0.9068 | 0.6369 |
|  |  | LightGBM | 0.7524 | 0.7436 | 0.7448 | 0.7524 | 0.9104 | 0.6286 |
|  | traditional classifier | SVM | 0.7333 | 0.7259 | 0.7265 | 0.7333 | 0.8817 | 0.6000 |
|  |  | GNB | 0.6259 | 0.5775 | 0.5991 | 0.6164 | 0.8315 | 0.4350 |
|  |  | Logistic | 0.7000 | 0.6878 | 0.6877 | 0.7000 | 0.8629 | 0.5500 |
|  |  | DT | 0.6931 | 0.6862 | 0.6876 | 0.6890 | 0.7680 | 0.5390 |
| SMOTETomek | integrated classifier | DF | **0.8155** | **0.8116** | **0.8212** | **0.8131** | **0.9243** | **0.7231** |
|  |  | RF | 0.7873 | 0.7808 | 0.7847 | 0.7873 | 0.9175 | 0.6810 |
|  |  | Bagging | 0.7508 | 0.7423 | 0.7475 | 0.7508 | 0.8669 | 0.6262 |
|  |  | XGBoost | 0.7603 | 0.7496 | 0.7532 | 0.7603 | 0.9016 | 0.6405 |
|  |  | LightGBM | 0.7690 | 0.7581 | 0.7649 | 0.7642 | 0.9095 | 0.6526 |
|  | traditional classifier | SVM | 0.7448 | 0.7390 | 0.7423 | 0.7413 | 0.8951 | 0.6169 |
|  |  | GNB | 0.6444 | 0.5848 | 0.6289 | 0.6444 | 0.8484 | 0.4667 |
|  |  | Logistic | 0.7259 | 0.7139 | 0.7162 | 0.7209 | 0.8769 | 0.5877 |
|  |  | DT | 0.7095 | 0.7034 | 0.7047 | 0.7095 | 0.7821 | 0.5643 |
| SMOTEENN | integrated classifier | DF | **0.9032** | **0.6664** | 0.6735 | **0.6844** | 0.8788 | **0.8242** |
|  |  | RF | 0.8903 | 0.6529 | 0.6366 | 0.6749 | 0.8442 | 0.8014 |
|  |  | Bagging | 0.8903 | 0.6247 | 0.5965 | 0.6578 | 0.8276 | 0.7994 |
|  |  | XGBoost | 0.8774 | 0.6150 | 0.5863 | 0.6475 | 0.8536 | 0.7753 |
|  |  | LightGBM | 0.8903 | 0.6241 | 0.5949 | 0.6571 | 0.8352 | 0.7990 |
|  | traditional classifier | SVM | 0.8839 | 0.6204 | 0.5933 | 0.6533 | **0.9320** | 0.7879 |
|  |  | GNB | 0.5935 | 0.5614 | 0.6746 | 0.6366 | 0.8823 | 0.4292 |
|  |  | Logistic | 0.8710 | 0.6110 | 0.5822 | 0.6444 | 0.8874 | 0.7643 |
|  |  | DT | 0.8258 | 0.6630 | **0.6780** | 0.6824 | 0.7990 | 0.7110 |

Note: Bold indicates the best value for each evaluation metric across the different models.

Supplementary Table S7. Repeated stratified 10-fold cross-validation performance of nine classifiers under the CoopLearning + SMOTEENN framework

| Classifier | | Accuracy | F1-score | Precision | Recall | MAUC | Kappa |
| --- | --- | --- | --- | --- | --- | --- | --- |
| traditional classifier | SVM | 0.866±0.060  (0.854,0.878) | 0.866±0.059  (0.854,0.877) | 0.866±0.058  (0.855,0.877) | 0.885±0.054  (0.875,0.896) | 0.970±0.024  (0.966,0.975) | 0.790±0.092  (0.772,0.808) |
|  | GNB | 0.892±0.057  (0.881,0.903) | 0.891±0.056  (0.880,0.902) | 0.895±0.052  (0.885,0.905) | 0.902±0.057  (0.891,0.913) | 0.968±0.029  (0.963,0.974) | 0.828±0.089  (0.811,0.846) |
|  | Logistic | 0.838±0.067  (0.825,0.851) | 0.841±0.064  (0.829,0.854) | 0.840±0.060  (0.828,0.852) | 0.878±0.053  (0.868,0.889) | 0.974±0.023  (0.969,0.978) | 0.752±0.098  (0.733,0.772) |
|  | DT | 0.897±0.061  (0.885,0.908) | 0.892±0.061  (0.880,0.904) | 0.896±0.058  (0.884,0.907) | 0.899±0.063  (0.887,0.912) | 0.940±0.040  (0.932,0.948) | 0.834±0.097  (0.815,0.853) |
| integrated classifier | RF | 0.900±0.049  (0.890,0.910) | 0.896±0.052  (0.886,0.906) | 0.898±0.050  (0.888,0.908) | 0.905±0.053  (0.895,0.915) | 0.979±0.017  (0.975,0.982) | 0.840±0.078  (0.825,0.855) |
|  | Bagging | 0.886±0.054  (0.876,0.897) | 0.884±0.054  (0.873,0.895) | 0.886±0.054  (0.875,0.897) | 0.895±0.052  (0.884,0.905) | 0.974±0.021  (0.970,0.978) | 0.819±0.084  (0.803,0.836) |
|  | XGBoost | 0.905±0.058  (0.894,0.917) | 0.904±0.057  (0.893,0.915) | 0.903±0.053  (0.893,0.914) | 0.918±0.055  (0.907,0.929) | 0.973±0.026  (0.968,0.978) | 0.850±0.090  (0.833,0.868) |
|  | LightGBM | 0.896±0.058  (0.885,0.908) | 0.895±0.057  (0.883,0.906) | 0.894±0.055  (0.883,0.905) | 0.907±0.056  (0.896,0.918) | 0.977±0.026  (0.972,0.982) | 0.835±0.091  (0.818,0.853) |
|  | DF | **0.915±0.049**  **(0.905,0.924)** | **0.913±0.049**  **(0.903,0.922)** | **0.912±0.047**  **(0.903,0.921)** | **0.924±0.048**  **(0.915,0.934)** | **0.982±0.018**  **(0.978,0.986)** | **0.864±0.077**  **(0.849,0.879)** |
| Friedman test *P-value* | | ＜0.001 | ＜0.001 | ＜0.001 | ＜0.001 | ＜0.001 | ＜0.001 |

Note: The data in the table are performance metrics on the test sets obtained from 10 repeated 10-fold cross-validation and are presented as mean ± standard deviation. Values in parentheses indicate the 95% confidence interval. Bold values indicate the best performance for each evaluation metric.
